# Health Professional vs Layperson Values and Preferences on Scarce Resource Allocation

**DOI:** 10.1001/jamanetworkopen.2024.1958

**Published:** 2024-03-12

**Authors:** Russell G. Buhr, Ashley Huynh, Connie Lee, Vishnu P. Nair, Ruby Romero, Lauren E. Wisk

**Affiliations:** 1 Division of Pulmonary and Critical Care Medicine, David Geffen School of Medicine at the University of California, Los Angeles; 2 Center for the Study of Healthcare Innovation, Implementation, and Policy, Health Services Research and Development, Greater Los Angeles Veterans Affairs Healthcare System, Los Angeles, California; 3 Department of Health Policy and Management, Fielding School of Public Health at the University of California, Los Angeles; 4 Clinical and Translational Science Institute Research Associates Program, University of California, Los Angeles; 5 University of California Irvine School of Medicine, Irvine; 6 Keck Graduate Institute School of Pharmacy and Health Sciences, Claremont, California; 7 David Geffen School of Medicine, University of California, Los Angeles; 8 Department of Medicine, Stanford University, Stanford, California; 9 Division of General Internal Medicine and Health Services Research, David Geffen School of Medicine at the University of California, Los Angeles

## Abstract

**Importance:**

COVID-19 prompted rapid development of scarce resource allocation (SRA) policies to be implemented if demand eclipsed health systems’ ability to provide critical care. While SRA policies follow general ethical frameworks, understanding priorities of those affected by policies and/or tasked with implementing them is critical.

**Objective:**

To evaluate whether community members and health care profesionals (HCP) agree with SRA protocols at the University of California (UC).

**Design, Setting, and Participants:**

This survey study used social media and community-partnered engagement to recruit participants to a web-based survey open to all participants aged older than 18 years who wished to enroll. This study was fielded between May and September 2020 and queried participants’ values and preferences on draft SRA policy tenets. Participants were also encouraged to forward the survey to their networks for snowball sampling. Data were analyzed from July 2020 to January 2024.

**Main Outcomes and Measures:**

Survey items assessed values and preferences, graded on Likert scales. Agreement was tabulated as difference in Likert points between expressed opinion and policy tenets. Descriptive statistics were tested for significance by HCP status. Free text responses were analyzed using applied rapid qualitative analysis.

**Results:**

A total of 1545 participants aged older than 18 years (mean [SD] age 49 [16] years; 1149 female participants [74%], 478 health care practitioners [30%]) provided data on SRA values and preferences. Agreement with UC SRA policy as drafted was moderately high among respondents, ranging from 67% to 83% across domains. Higher agreement with the interim policy was observed for laypersons across all domains except health-related factors. HCPs agreed more strongly on average that resources should not be allocated to those less likely to survive (HCP mean, 3.70; 95% CI, 3.16-3.59; vs layperson mean, 3.38; 95% CI, 3.17-3.59; *P* = .002), and were more in favor of reallocating life support from patients less likely to those more likely to survive (HCP mean, 6.41; 95% CI, 6.15-6.67; vs layperson mean, 5.40; 95% CI, 5.23-5.58; *P* < .001). Transparency and trust building themes were common in free text responses and highly rated on scaled items.

**Conclusions and Relevance:**

This survey of SRA policy values found moderate agreement with fundamental principles of such policies. Engagement with communities affected by SRA policy should continue in iterative refinement in preparation for future crises.

## Key Points

**Question:**

What are the opinions and values of key informants among both laypersons and health care professionals regarding the design and implementation of scarce resource allocation (SRA) policy under crisis standards of care?

**Findings:**

This survey study of 1545 participants found moderate agreement with policy tenets from the University of California SRA policy. Health care professionals agreed more strongly with both prioritizing resources for and reallocating existing resources to those most likely to survive.

**Meaning:**

These findings suggest common SRA policy tenets are broadly acceptable to both laypersons and health care professionals, though substantial disagreement on logistics of implementation remains.

## Teaser

This survey study evaluates whether community members and health care professionals agree with scarce resource allocation protocols developed in a US healthcare system.

## Introduction

The COVID-19 pandemic caused unprecedented strains on health systems. Significant concerns about resource shortages motivated assessment of existing policies regarding scarce resource allocation (SRA) that would be put into place when the demand for resources eclipsed the ability to provide standard care.

Although fundamental ethical principles in health care are consistent (ie, beneficence, justice, autonomy, respect for persons, and nonmaleficence), SRA policies vary in how these domains are prioritized.^1,2^ Limited empirical evidence on SRA design and logistics existed early in the pandemic when SRA policies were being developed. Rapid feedback on perspectives of both the general public^3,4^ and, crucially, health care professionals (HCPs)^5^ was needed to inform policy makers.

The University of California convened a multicampus Critical Care Bioethics Working Group to develop guidance on SRA policy under crisis standards of care in March 2020, representing each campus with a medical center, collectively known as UC Health.^6^ Other jurisdictions also released guidance during this period with congruent principles but varying decision algorithms.^7,8^

Ideally, public and HCP input would be sought during the drafting of policy guidance to ensure that key informant values and preferences are incorporated. While real-time collaborative efforts to draft acceptable policy would have been preferable, COVID-19 presented unique challenges, including the rapid escalation of the pandemic necessitating swift development and stay-at-home policies that made convening consensus-making groups difficult. When unable to obtain direct input during crafting of guidance, we must instead seek feedback on drafted policies and treat them as living documents, subject to modification as needed.

With these facts in mind, UC Health recognized the need for community feedback and sponsored a community-centered survey for rapid deployment, known as the Understanding Community Considerations, Opinions, Values, Impacts, Decisions, or UC-COVID Study.^9^ Its goals were to elucidate preferences and values for SRA policies, including the logistics of policy implementation and factors associated with prioritization schema, to identify potential areas of conflict to garner community buy-in,^3,4,5^ and to inform ongoing development and refinement of SRA policies.^2,10,11,12,13,14^

We detail the UC-COVID findings in this manuscript, particularly to compare values and preferences for such policies between laypersons who may be subject to these decisions vs HCPs who may be tasked with implementing these decisions during a crisis to identify alignment and disagreement between these critical groups to provide policy makers information on areas in need of refinement.

## Methods

### Survey Design and Measures

We drafted and deployed an internet-based survey to engage community opinions on the design, content, and dissemination of SRA policy, as previously described.^9^ Survey flow was stratified by self-identified HCP status, such that HCPs received some different questions than non-HCPs (hereafter referred to as laypersons), particularly surrounding technical details of the SRA policy. Because of concerns about historic and ongoing inequities in the experience of minoritized groups receiving health care, we collected self-reported identifiers including race, ethnicity, sexual orientation, and gender identity to better understand where priorities may differ across groups. Race was operationalized using US Census categories as select all that apply options and an other option with free text and collected as self-report. We collected gender identity as self-report with a free text option for other including gender nonconforming or nonbinary. Survey questions were deployed on REDCap version 10.6.14 (Vanderbilt University)^15,16^ initially. They were subsequently professionally translated (International Contact) into Spanish, Korean, Simplified Chinese, Vietnamese, and Tagalog (the top 5 non-English spoken languages in California) and migrated to Qualtrics XM (Qualtrics, LLC) for multilingual support, which was not available in REDCap. This study was approved by the UCLA institutional review board. Electronic informed consent was obtained from all participants. The American Association for Public Opinion Research (AAPOR) reporting guidelines were used in the design and reporting of this survey.

### Recruitment and Sampling of Participants

The study was disseminated through snowball sampling using social media platforms, including Facebook, Twitter, Instagram, and LinkedIn, and direct recruitment through partner organizations (eg, Chronic Obstructive Pulmonary Disease Foundation, Taking Control of Your Diabetes, Pulmonary Hypertension Association, Vietnamese Cancer Foundation, and Altamed) and professional societies (eg, California Thoracic Society, American Thoracic Society, and Society for General Internal Medicine). Although our recruitment strategy primarily focused on groups in California (71% of our initial sample), eligibility was not restricted by location, and all adults (aged ≥18 years) were eligible. We recruited from May to September 2020.

### Assessment of Values on SRA

Participants were asked a series of questions about logistics for the implementation of SRA policies, including who should be responsible for SRA policy development, how these policies should be disseminated to the public and to patients being affected by SRA decisions, and what protections should be afforded HCPs who must deploy SRA policies, rated on a 10-point Likert scale (1, “I strongly disagree” to 10, “I strongly agree”) with no neutral midpoint to force participants to lean one direction or the other. We asked about their opinion on whether specific health-related and social factors should be used to make determinations about life support allocation. Assessments of values regarding life support allocation, including medical and non–health-related factors, were made on a 9-point Likert scale, where 1 was “Should be much less likely to get life support,” 5 was “Should not influence one way or the other,” and 9 was “Should be much more likely to get life support.” A full accounting of items grouped by domain is found in eTable 1 in Supplement 1.

### Defining Agreement With SRA Policy Tenets

Items were scored for concurrence with SRA policy tenets by referencing against the published policy designed and approved by UC Health.^6^ For each question, we determined if the question corresponded with the UC policy (for example, a tenet of the UC policy was to try to save the greatest number of lives possible) and scored respondents’ agreement based on a percentage alignment with the ideal score (ie, for items that mapped to UC policy drafted tenets, respondents with a score of 10 [for logistics] or 9 [for all other scales] were deemed to have 100% agreement if the policy matched this opinion; all other scores were compared against the 100% agreement such that the opposite score [eg, a value of 1] would be given 0% agreement). For questions in which the answer most aligning with the UC policy was a middle score of no influence (ie, 5 on a scale of 1 to 9), a score of 5 had 100% agreement and scores of either 1 or 9 had 0% agreement.

### Statistical Analysis

Descriptive analyses of survey responses comparing demographic groups of interest were conducted where continuous responses were assessed using *t* tests and categorical variables were compared by χ^2^ tests or Fisher exact tests when appropriate. Using the agreement level defined above, we fit a series of fractional regression models with probit link to estimate the outcomes of respondent characteristics on proportion of agreement with SRA policy tenets.^17^ Statistical analyses were conducted in SAS version 9.4 (SAS Institute) and Stata version 18.0 (StataCorp), and violin plots were generated in R Studio version 2023.12.0+369 (R Project for Statistical Computing). All *P* values were 2-sided with an α of .05. Data were analyzed from July 2020 to January 2024.

#### Qualitative Analysis

Free-text response fields were used to allow participants to provide further context on their perspectives of SRA policies. The comments were exported for analysis and responses were coded using NVIVO version 1.3.2 (QSR International). We used a combination deductive-inductive rapid qualitative analytic approach,^18,19^ where a conceptual framework was derived in parallel to the SRA domains, with open coding of further inductive themes. Three analysts (A.H., C.L., and V.P.N. under supervision of R.G.B. and L.E.W.) independently read and coded each transcript. From these codes, themes emerged and were ranked in importance to the respondents based on frequencies of appearance.

#### Missing Data

Multiple imputation by fully conditional specification, with 10 imputed data sets, was used to impute missing items within a scale when a participant answered at least 1 question in each scale (eTable 1 in Supplement 1). Self-reported understanding of SRA policies and all prior SRA sections were included in each section-specific imputation equation to improve the specification. As results were consistent between imputed and nonimputed data sets (eTable 2 in Supplement 1), we used the imputed data in sensitivity analysis and report the data as observed (nonimputed) as the main analysis.

## Results

### Participant Characteristics

Of the 1971 study participants, 1545 (78%) completed any of the SRA questions. Of these, 478 (31%) self-identified as HCPs, 1149 (74%) identified as female, and the mean (SD) age was 49 (16) years (Table 1). The majority of the participants completed the survey in English (1483 participants [96%]). Compared with the adult population of California, study participants were more likely to identify as female, less likely to identify as Hispanic, and more likely to report having a bachelor’s degree.

**Table 1.** Demographic Characteristics of Participants

Characteristic	Participants, No. (%)			
	All participants (N = 1545)	Laypersons (n = 1067)	Health care professionals (n = 478)	*P* value^a^
Age, mean (SD), y	49 (16)	51 (16)	45 (14)	<.001
Gender				
Female	1149 (74.4)	801 (75.1)	348 (72.8)	.36^c^
Male	378 (24.5)	251 (23.5)	127 (26.6)	
Other/prefer not to answer^b^	18 (1.2)	15 (1.4)	3 (0.6)	
Sexual orientation				
Straight/heterosexual	1148 (74.3)	807 (75.6)	341 (71.3)	<.001
Gay/lesbian	91 (5.9)	58 (5.4)	33 (6.9)	
Bisexual	87 (5.6)	66 (6.2)	21 (4.4)	
Other/not sure^b^	85 (5.5)	69 (6.5)	16 (3.3)	
Not answered	134 (8.7)	67 (6.3)	67 (14.0)	
Racial identity^d^				
White	1233 (79.8)	884 (82.8)	349 (73.0)	<.001
Black	74 (4.8)	46 (4.3)	28 (5.9)	.19
American Indian/Alaska Native	1516 (98.1)	1045 (97.9)	471 (98.5)	.54^c^
Asian/Pacific Islander	206 (13.3)	116 (10.9)	90 (18.8)	<.001
Other	22 (1.4)	16 (1.5)	6 (1.3)	.82^c^
Latin/Hispanic ethnicity	154 (10.0)	91 (8.5)	63 (13.2)	.01
Marital status				
Married/living with partner	1089 (70.5)	731 (68.5)	358 (74.9)	.03
Widowed/divorced	176 (11.4)	133 (12.5)	43 (9.0)	
Never married	280 (18.1)	203 (19.0)	77 (16.1)	
Have children	477 (30.9)	290 (27.2)	187 (39.1)	<.001
Bachelor’s degree or higher	1329 (86.0)	889 (83.3)	440 (92.1)	<.001
Employment status				
Working currently	1016 (65.8)	635 (59.5)	381 (79.7)	<.001
Have job, not working	109 (7.1)	71 (6.7)	38 (7.9)	
Retired or student	248 (16.1)	211 (19.8)	37 (7.7)	
Not working currently	172 (11.1)	150 (14.1)	22 (4.6)	
Self-reported essential worker status	585 (37.9)	193 (18.1)	392 (82.0)	<.001
Currently have health insurance	1489 (96.4)	1022 (95.8)	467 (97.7)	.32
Political views				
Pretty conservative	61 (3.9)	39 (3.7)	22 (4.6)	<.001
More conservative than liberal	149 (9.6)	106 (9.9)	43 (9.0)	
In the middle	226 (14.6)	151 (14.2)	75 (15.7)	
More liberal than conservative	278 (18.0)	190 (17.8)	88 (18.4)	
Pretty liberal	634 (41.0)	477 (44.7)	157 (32.8)	
Not political	66 (4.3)	40 (3.7)	26 (5.4)	
Not answered	131 (8.5)	64 (6.0)	67 (14.0)	

^a^ *P* values represent Welch *t* test for continuous variables and χ^2^ tests for categorical variables unless otherwise stated.

^b^ Response categories combined to suppress low cell counts.

^c^ Calculated using Fisher exact test.

^d^ Question framed as select all that apply; percentages may add to more than 100%, and each line represents difference in proportions selecting that respective race as one of their identifiers.

Of those who identified as HCPs, 126 (25%) were physicians; 46 (9%) nurse practitioners, physician assistants, and clinical nurse specialists; 125 (25%) registered nurses; and the remainder allied health professionals including rehabilitation services, respiratory therapists, social workers, chaplains, and pharmacy staff. A total of 392 health care professionals (82%) self-reported essential worker status. Among physicians, 24 physician respondents (19%) reported subspecialization in critical care medicine.

### Values on SRA Policy Logistics

**Figure 1.**
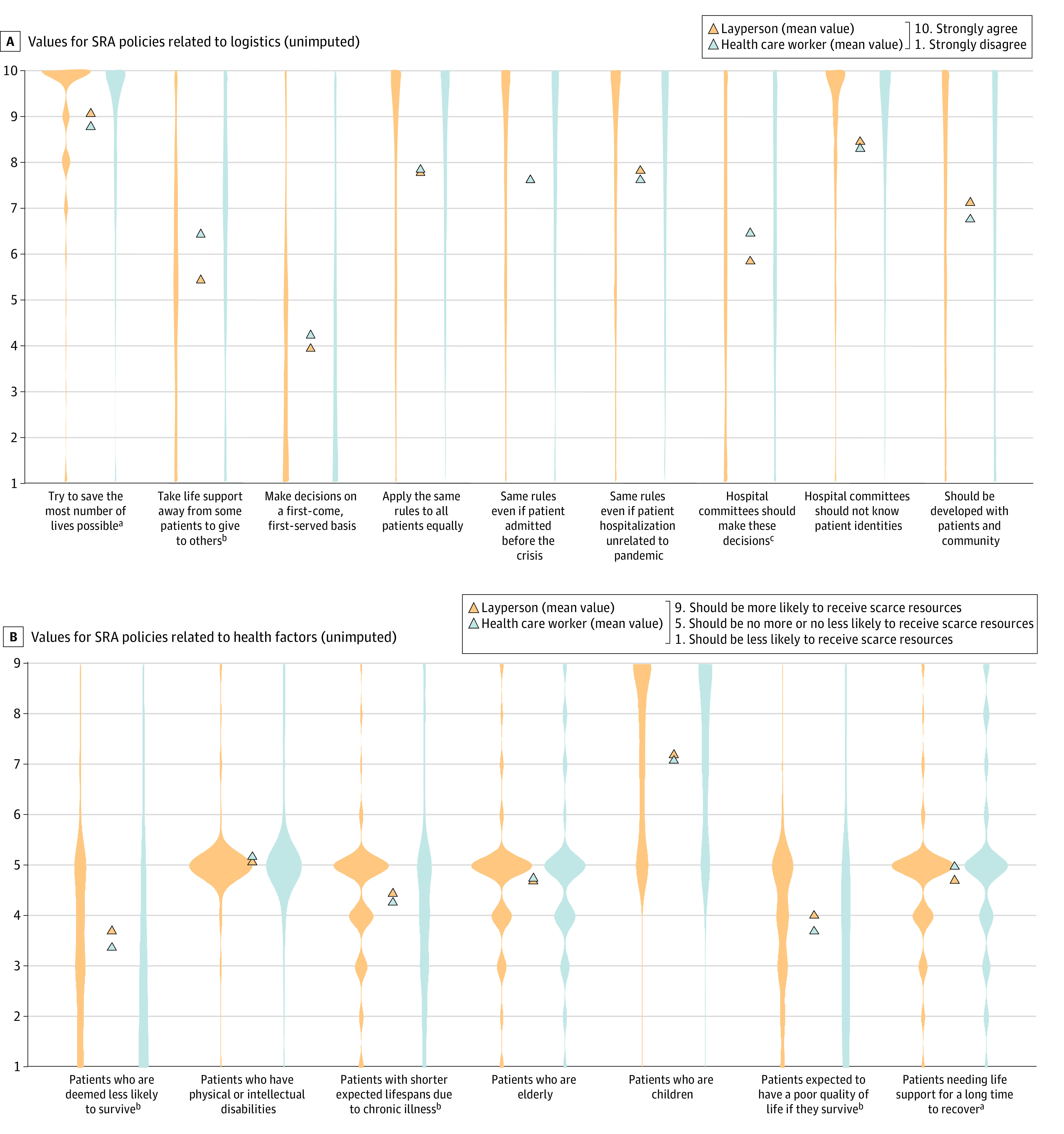
Responses to Scarce Resource Allocation (SRA) Questions by Health Care Worker Status Plots demonstrate distribution of responses. The blue strips represent self-identified health care workers while the orange strips represent laypersons, with the triangles between strips showing the mean response for each question for respondent type. ^a^Indicates *P* < .05. ^b^Indicates *P* < .001. ^c^Indicates *P* < .01.

HCPs and laypersons felt that SRA policy should aim to save the most lives possible, with a significantly higher 0.29 point mean agreement score among laypersons vs HCPs (mean, 9.04; 95% CI, 8.93-9.16; vs 8.76; 95% CI, 8.56-8.92; *P* = .005) (Figure 1A). Agreement that life support should be taken away from people less likely to survive to enable its use by people more likely to survive was significantly higher among HCPs vs laypersons (1.01 points difference; mean, 6.41; 95% CI, 6.15-6.67; vs 5.40; 95% CI, 5.23-5.58; *P* < .001). We observed high concurrence that the SRA rules should apply to everyone, regardless of whether hospitalization was related to the cause of the crisis, and regardless of whether the patients were admitted before the crisis was declared; this did not differ between groups. Both HCPs and laypersons felt strongly that the committees should be created to make SRA decisions and blinded to the identity of the patients for whom SRA decisions were being made. Laypersons indicated stronger sentiment toward involving patients in SRA policy development decisions than HCPs did (mean, 7.10; 95% CI, 6.92-7.28; vs 6.74; 95% CI, 6.44-7.03; *P* = .02).

### Values on SRA Policy Health-Related and Social Factors and Exceptions

HCPs and laypersons felt that those less likely to survive should be deprioritized to receive life support measures, but HCPs significantly more so than laypersons (mean, 3.70; 95% CI, 3.16-3.59; vs 3.38; 95% CI, 3.17-3.59; *P* = .002) (Figure 1B). Compared with laypersons, HCPs deprioritized those with a poor expected quality of life if they survived (mean, 3.70; 95% CI, 3.51-3.89; vs 4.02; 95% CI, 3.92-4.11; *P* < .001). Neither group felt that disability status or age should significantly influence allocation of life sustaining measures, which did not differ between the 2 groups (eFigure 1 in Supplement 1). Laypersons were more likely to agree with special exceptions to SRA policies than HCPs across the board, however moderate prioritization for critical resources (mean score >7) were observed only for pregnant persons in the third trimester and front-line HCPs (eFigure 2 in Supplement 1).

### Communication and Dissemination Preferences

Laypersons felt more strongly that SRA policies be made public compared with HCPs (mean, 8.42; 95% CI, 8.28-8.57; vs 8.09; 95% CI, 7.84-8.33; *P* = .008) (eTable 2 in Supplement 1). Neither group felt that disclosure should be limited to patients admitted in critical condition (mean, 4.96; 95% CI, 4.65-5.27 for HCPs; vs 5.08; 95% CI, 4.86-5.30 for laypersons; *P* = .53). Both groups agreed that they would consider SRA policies when choosing a hospital in which to seek care with laypersons feeling more strongly than HCPs (mean, 7.71; 95% CI, 7.54-7.88; vs 7.41; 95% CI, 7.06-7.66; *P* = .03). When HCPs were asked about how comfortable they were explaining policies to their own patients, weak agreement was noted for both verbal (mean [SD], 6.64 [2.88]) and written (mean [SD], 6.68 [2.89]) explanations. At the same time, laypersons moderately agreed that they would feel more at ease if their practitioner provided them either a verbal (mean [SD], 9.06 [2.32]) or written (mean [SD], 7.80 [2.50]) explanation of policies.

### Agreement With UC Health SRA Policy Tenets

**Figure 2.**
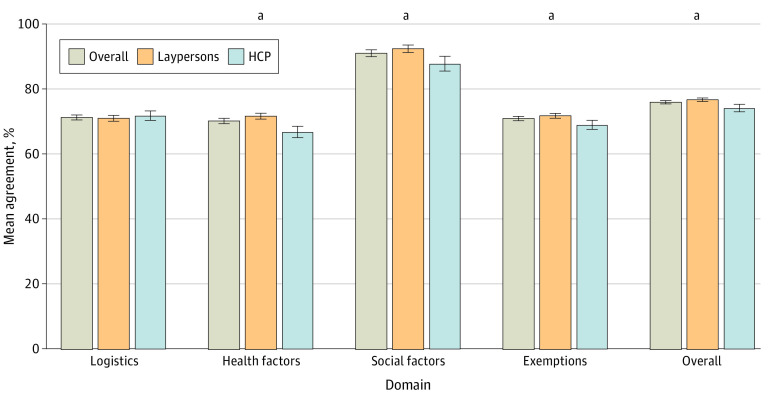
Proportion of Respondents Expressing Agreement With University of California Scarce Resource Allocation Policy Tenets as Written by Domain For each domain, participants were included if they responded to at least 2 items from the domain. For overall agreement, participants were included if they responded to at least 1 item from each domain. HCP indicates health care personnel. ^a^Indicates *P* < .001 between groups.

Raw agreement was aggregated across domains, stratified by HCP status, and high among all respondents, ranging from 67% to 83% (Figure 2). On items regarding the logistics of how SRA should be implemented, agreement with policy as designed was similar among HCPs (mean [SD], 71.9% [15.5%]) when compared with laypersons (mean [SD], 71.1% [14.6%]). Interestingly, laypersons’ responses were significantly more aligned with the SRA policy than HCPs with regards to health factors, social factors, and exemptions. Taken together, the values of the survey respondents reflected an alignment of 76.0% with UC Health SRA policy, with the mean agreement slightly higher for laypersons than HCPs (76.8%; 95% CI, 76.3%-77.3%; vs 74.2%; 95% CI, 73.1%-75.4%; *P* < .001) (eTable 3 in Supplement 1). In sensitivity analyses, no differences in sign nor significance were observed when comparing as-observed vs imputed values for agreement across domains. Additionally, California respondents rated agreement slightly higher than overall respondents, but proportion of agreement did not differ by HCP vs layperson within California respondents (eTable 3 and eTable 4 in Supplement 1).

**Figure 3.**
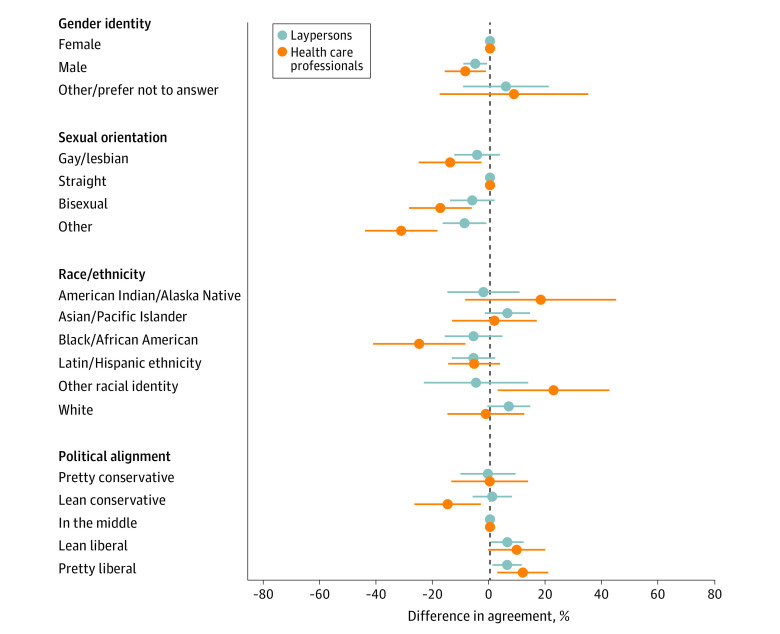
Differences in Agreement With Exemptions to Scarce Resource Allocation Policy by Respondent Characteristics, Stratified by Health Care Professional Status Fractional model additionally controlling for age, marital status, employment, education, households with children, and essential worker status.

When stratified by HCP group membership in our regression models, men, Black, Latin or Hispanic respondents, and those from sexually minoritized groups had lower mean adjusted agreement percentages with SRA policy tenets, both overall (Figure 3) and by subdomains (eFigure 3, eFigure 4, eFigure 5, and eFigure 6 in Supplement 1). Conversely, those who expressed more liberal political alignment were significantly more likely to agree with SRA policy tenets even after adjusting for other sociodemographic factors.

### Qualitative Responses

Free text responses regarding SRA policy, which were given spontaneously by respondents, were recorded for 459 of the participants, including 79 HCPs (14%) and 380 lay respondents (27%). We found 4 main recurring themes (Table 2). The most prevalent recurrent theme underscored the importance of having interested parties involved in decision making. Several respondents noted that both those affected by and those implementing such policies should be involved together in policy derivation and implementation. The next major theme surrounded trust and transparency. Respondents were somewhat divided on their opinion of the trustworthiness of who would make such decisions. A common response was that while individuals trusted their own physicians, they would not necessarily trust administration, government, or practitioners with whom they did not have prior relationships. Respondents were also mixed about transparency and public availability of such a policy. While some felt that transparency would maximize accountability of health systems, others felt that publicizing policies would enable patients with privilege and resources to pick and choose their hospitals based on whether they felt a policy would put them at a disadvantage to receive life-sustaining care. The next most prevalent theme related to equity and justice, with respondents highlighting concerns about equity implications and how SRA policies may disproportionately impact marginalized groups and exacerbate structural inequities. Finally, multiple responses mapped to an overall theme of the challenges of conceptualizing a widely accepted policy no matter what approach was taken. Taken in tandem with the quantitative results, these comments demonstrated the complexity, nuance, and even cognitive dissonance associated with respondents’ values and preferences.

**Table 2.** Thematic Analysis of Free Text Responses With Representative Examples

Theme	Example Responses
1: Interested party involvement in decision making. Participants regularly highlighted wanting expert input from frontline providers and that community members should have input on local policies.	“The option of doctor vs committee making the decisions is a tricky one and something that could vary quite a bit between locations with regards to which option is preferable. While a more personal relationship with the patient (even a brief one) can bring emotion into the equation on the part of the provider and can cloud decision making, committees, inevitably, slow things down.”
	“Policies should be put in place by the doctors and nurses working on the frontlines, no one that is not daily exposed to this should have a say. Paper pushers should definitely not have a say.”
	“Guidelines should be developed by medical professionals and governments, but input from family and community and the patients’ own preferences should be considered before action is taken (or not taken).”
2: Trust and transparency. Participants were divided over their opinion of the trustworthiness of hospital administration, individual health care professionals, and federal policy makers regarding transparency and fairness, and whether such policies should be publicly available.	“I worry less about the doctors being honest and transparent and more about the hospitals doing so. Many hospital administrations...seem to fear transparency and accountability because they are afraid of liability and politics.”
	“If policies are truly transparent and clear to all (patients as well as health providers) then the laws should protect both equally, if a medical provider does not obey the law/policy then they should be held accountable. As in any case of malpractice or negligence, the case must be proven and the right decisions made as to intent.”
	“Letting people know what the policy is would allow them to cherry pick the places in which they are most likely to survive, only if they are privileged enough to not have a condition putting them at a lower chance of getting a ventilator. Also, knowing the policy wouldn’t make people feel more at ease, because either way (random or strategic) people will be upset with the outcome if they or their family member is not chosen.”
3: Equity and justice. Participants emphasized equity implications in scarce resource allocation policies regarding impacts toward marginalized groups and present structural biases.	“Communities of color know, science has always been racialized and medicine/health care weaponized against us—how can we expect anything different now.”
	“Some of these are very hard to answer. Regarding race, for example, I wish it didn’t matter, but I believe that there are implicit decisions made because of race. And I want to say that perhaps racial minorities should get more care because they are more likely to have undetected health problems so would need more care to survive. But I also am not sure that undetected health problems could be used to make rules about medical decisions.”
	“No policy is acceptable unless it is exposed to the full scrutiny and activism of affected members of the communities described in the policy. We went through this in the American response to AIDS and we now know that full buy-in and cooperation between health professionals and affected communities advances the state of care and research. (I trust people I select to be honest as practitioners, but those trust effects do not scale upward to for-profit insurers, hospital systems of any size or profit status, or professional associations.)”
4: Overall challenges in forming an acceptable scarce resource allocation policy. Participants acknowledged the difficulty in conceptualizing an acceptable policy.	“I think having a decision tree helps, and to have those shaped by committees that include the community. However, I have trouble envisioning a working system where individual doctors aren’t making these emergency decisions. And once you vent someone, it’s a bigger decision to [take someone off of mechanical ventilation] them than not to have [put them on mechanical ventilation] in the first place.”
	“These kinds of decisions are hard but they need to be made at times. Better that physicians make these decisions on an individual basis informed by general policies (with the support of ethics committees and such when necessary) than allow anyone else to do so for reasons unbound by professional medical ethics.”
	“It is difficult, because the Hippocratic oath tells us to first do no harm, and this does ride the line between saving lives and having to choose to do harm to and to allow to possibly die. I think that I probably would have great difficulty implementing these measures if I was working inpatient.”
	“I think this is an extremely complex subject, including that our country and health care system has failed to provide equitable care through access, quality, and policies. Additionally, the ADA means that we cannot (and should not!) decide someone is less deserving of care because they have a disability or have what some consider a “lower quality of life.” Essential workers (not just in health care! include the grocery store workers, garbage collectors, bus drivers, grocery delivery service, etc.) have a higher risk of contracting the novel corona virus, and because they’re serving the country. Additionally, the lower wage essential workers are disproportionately people of color, and POC are already at higher risk due to social determinants of health and the racism that drives them. Again: failing system. In working with patients (as a student) I’ve found that the case you read on paper often doesn’t match the health case you see in front of you, yet I wouldn’t want [an] individual (and the internal biases that sway us all) to be making these decisions. Yet hospitals and algorithms decide based on the health profile written about a person, which is also biased against people with disabilities or the possibility of future disability and that’s bloody wrong. So, I’ve been wrestling with these issues. I don’t know the answers, but I do know some of the problems with how the answers are currently being constructed.”
	“Good luck with this, invariably 50% of people will be [mad] at whatever decisions are made.”

## Discussion

In this survey surrounding the design and implementation of SRA policies, we found that values and preferences were highly aligned with the UC Health SRA policy at approximately 70% or higher agreement by domain with significant, albeit small magnitude, differences between laypersons and HCPs. This study adds to the limited literature on opinions of people potentially affected by SRA policy, comparing the values of patients who may seek hospitalization during a crisis and those of HCPs tasked with providing care and implementing such policies. We observed that HCPs held more stringent views on critical care resource allocation than laypersons, being more likely to deprioritize those with both lower expected in-hospital survival, and lower general life expectancy, and were less likely to favor exceptions to SRA policies.

### Agreement With Underlying Ethical Principles

The UC Policy, like many SRA policies, prioritizes saving the most lives possible and otherwise random allocation within prioritization tiers for those deemed to have comparable survivability, with some additional considerations for instrumental utility (eg, prioritizing essential HCPs who may reenter the workforce). We observed strong agreement among respondents with this schema, somewhat in contrast to results from Korea, where reciprocity and instrumental value were placed significantly lower in order of priority,^20^ but well aligned with other investigations.^4,21,22^

We also observed findings consistent with others’ in that factors not related to health status or sociodemographics should generally not affect resource allocation.^23^ While advanced age was felt to be a reason to deprioritize ventilators in some studies,^23,24,25,26^ our findings showed that age should not affect decisions one way or the other, while the Department of Health and Human Services Office of Civil Rights precludes resource allocation based directly on age.^27^

We found fairly high alignment between laypersons and HCPs across topics. The largest discrepancy we noted was whether life support should be withdrawn from a patient less likely to survive to provide it to a patient more likely to survive, where HCPs had a full point higher agreement, consistent with prior data from Japan by Norisue and colleagues.^28^ This finding demonstrates consistent cognitive dissonance across contexts and populations; while respondents favored prioritizing survival, they were often less comfortable with reallocating existing life-sustaining resources. Studying the optimal communication methods needed to build consensus around this challenging topic should be an ongoing priority for study in this area, as without the ability to reallocate resources, such policies will encounter barriers and become de facto first-come first-served systems.

### Desire for Transparency and Fairness

We observed strong general sentiment toward transparency in the process of deriving, implementing, and executing such policies for scarce resource allocation. The UC Critical Care Bioethics Working Group, like many other bodies,^29,30^ was tasked with a very short timeline to design SRA policy in this analysis, and was unable to fully engage the community in the policy drafting given the rapidly encroaching crisis.^31,32^ Informing the public about dilemmas faced in developing SRA policies increased agreement with their legitimacy^33^ and reinforces the moral responsibility that the medical establishment and community have to one another.^34^ HCPs have expressed moral distress at the idea of implementing policies,^35^ which we also found, and bolstering frontline practitioner support through involving them in decision-making is crucial to successfully navigating such a crisis that would necessitate SRA decisions.

Shared decision-making around life sustaining treatment during critical illness is best practice for patient-centric care in intensive care units.^36^ Discrepancies between patient or surrogate and HCP impressions of survivability of critical illness or injury can be significant and result in potentially inappropriate life support. This gap is navigated through careful communication, value finding, and bilateral education: surrogates and patients to practitioners on treatment preferences, and practitioners to patients and surrogates about expected outcomes.^37^ During a crisis, however, the urgency to make triage decisions as patient demand rapidly eclipses supply of resources may undermine the ability for prolonged deliberation on treatment planning around life-sustaining measures. As such, engagement of a broad variety of key informants during policy development is optimal.

### Concerns Surrounding Equity and Justice

Continued concern about perpetuating or exacerbating inequalities in health care is warranted.^38^ Exploration of the potential for racial bias in these algorithms has been undertaken in limited degree, demonstrating a possible difference in model performance across racial groups.^39^ Ongoing concerns about how minoritized communities may be disproportionately affected are raised throughout recent literature and our findings continue to demonstrate lack of consensus in both SRA policy and among critical care resource use in general.^40,41,42,43^ While determination of how racial bias should be mitigated was not directly addressed in our study, we did find that both HCPs and laypersons felt less strongly about deprioritizing people with a shorter expected lifespan if a patient were also a member of a minoritized group, living with a disability, or living in poverty (eTable 2 in Supplement 1). With multiple policies using organ failure acuity scores whose validity across populations and conditions have been called into question,^44,45^ including that of UC Health, empirical evidence on optimizing SRA algorithms is sorely needed. Our findings further reinforce an unanswered research question of how to operationalize an algorithm that accounts for social determinants of health in allocation decisions.^46^

### Limitations

This study had limitations. The survey instruments we used were mapped to the tenets of UC Health SRA policy, but the participants were not shown the policy itself; therefore, our findings should be interpreted as illustrative of principles rather than an endorsement of this specific policy. We did not include reverse-scaled items due to survey length, and therefore are limited in internal consistency testing, although our free text responses were aligned with the patterns in our quantitative responses. Additionally, there are not any standard accepted minimum important difference thresholds reported for these types of items; therefore, while statistically significant results are described, the real-world impact of these differences are more challenging to measure.

This study recruited through community-based participation using snowball sampling and therefore was not fully representative of the US population. While perhaps less generalizable, this method of recruitment is known to generate valuable insights and may be particularly useful during a pandemic when recruitment to studies is more challenging^9,47^; nearly all existing empirical work in this area relies on online surveys.^4^ Our sample was overrepresented with participants who identified as highly educated, female, or living with a chronic medical condition. While we sought to have a maximally representative sample by offering the survey in the 5 most spoken languages in California and partnering with community organizations, further outreach to underrepresented and minoritized communities could improve generalizability.

## Conclusion

In this web-based survey of values surrounding SRA policy, we found moderate agreement with fundamental principles of such policies. Across a diverse group of HCPs and layperson respondents, our results highlighted that the derivation of a policy generally acceptable to both those affected by and those tasked with implementing triage allocation policies during a crisis is achievable, with strong agreement between our survey respondents and the currently written UC Health SRA policy. While UC Health’s policy is well-aligned with public opinion as observed in these results, our findings underscore the need to engage interested parties in policy drafting to ensure values are maximally represented, and to ensure that algorithms are developed with transparency and equity in mind. Potential shortcomings of SRA policies, particularly given their rapid design during the early COVID-19 crisis, should be further explored and community partnerships fostered to ensure maximal acceptability during the revision process.

## Notes

Supplement 1. **eTable 1. **Questions From the UC-COVID Survey With Scaling and Sampling Universe **eFigure 1. **Values for SRA Policies Related to Social Factors (Unimputed) **eFigure 2. **Values for Use of Exemptions in SRA Design **eTable 2. **Response Scores Comparing Layperson and HCP by Item, Unimputed Responses **eTable 3. **Nonimputed (All Respondent) vs. Mean Imputed Respondent and Unimputed California-Only Responses for Agreement Ratings With UC SRA Policy Domains as Drafted **eTable 4. **Response Scores Comparing Layperson and HCP by Item, Restricted to California Respondents **eFigure 3. **Differences in Agreement With Logistics of SRA Policy Implementation by Respondent Characteristics, Stratified by HCP Status **eFigure 4. **Differences in Agreement With Use of Health Factors in SRA Policy Design by Respondent Characteristics, Stratified by HCP Status **eFigure 5. **Differences in Agreement With Use of Nonhealth Social Factors in SRA Policy Design by Respondent Characteristics, Stratified by HCP Status **eFigure 6. **Differences in Agreement With Exemptions to SRA Policy by Respondent Characteristics, Stratified by HCP Status

Supplement 2.
Data Sharing Statement
