# Supplementary material for: Health Professional vs Layperson Values and Preferences on Scarce Resource Allocation
Source: JAMA Netw Open. 2024 Mar 12;7(3):e241958. doi: 10.1001/jamanetworkopen.2024.1958 (PMC10933708; doi:10.1001/jamanetworkopen.2024.1958)
Supplement: Supplement 1. — eTable 1. Questions From the UC-COVID Survey With Scaling and Sampling Universe eFigure 1. Values for SRA Policies Related to Social Factors (Unimputed) eFigure 2. Values for Use of Exemptions in SRA Design eTable 2. Response Scores Comparing Layperson and HCP by Item, Unimputed Responses eTable 3. Nonimputed (All Respondent) vs. Mean Imputed Respondent and Unimputed California-Only Responses for Agreement Ratings With UC SRA Policy Domains as Drafted eTable 4. Response Scores Comparing Layperson and HCP by Item, Restricted to California Respondents eFigure 3. Differences in Agreement With Logistics of SRA Policy Implementation by Respondent Characteristics, Stratified by HCP Status eFigure 4. Differences in Agreement With Use of Health Factors in SRA Policy Design by Respondent Characteristics, Stratified by HCP Status eFigure 5. Differences in Agreement With Use of Nonhealth Social Factors in SRA Policy Design by Respondent Characteristics, Stratified by HCP Status eFigure 6. Differences in Agreement With Exemptions to SRA Policy by Respondent Characteristics, Stratified by HCP Status [file jamanetwopen-e241958-s001.pdf]

Supplemental Online Content

Buhr RG, Huynh A, Lee C, Nair VP, Romero R, Wisk LE. Health professional vs layperson values and preferences on scarce resource allocation. *JAMA Netw Open*. 2024;7(3):e241958. doi:10.1001/jamanetworkopen.2024.1958

eTable 1. Questions From the UC-COVID Survey With Scaling and Sampling Universe
eFigure 1. Values for SRA Policies Related to Social Factors (Unimputed)
eFigure 2. Values for Use of Exemptions in SRA Design
eTable 2. Response Scores Comparing Layperson and HCP by Item, Unimputed Responses
eTable 3. Nonimputed (All Respondent) vs. Mean Imputed Respondent and Unimputed California-Only Responses for Agreement Ratings With UC SRA Policy Domains as Drafted
eTable 4. Response Scores Comparing Layperson and HCP by Item, Restricted to California Respondents
eFigure 3. Differences in Agreement With Logistics of SRA Policy Implementation by Respondent Characteristics, Stratified by HCP Status
eFigure 4. Differences in Agreement With Use of Health Factors in SRA Policy Design by Respondent Characteristics, Stratified by HCP Status
eFigure 5. Differences in Agreement With Use of Nonhealth Social Factors in SRA Policy Design by Respondent Characteristics, Stratified by HCP Status
eFigure 6. Differences in Agreement With Exemptions to SRA Policy by Respondent Characteristics, Stratified by HCP Status

This supplemental material has been provided by the authors to give readers additional information about their work.

© 2024 Buhr RG et al. *JAMA Network Open.*

eTable 1. Questions From the UC-COVID Survey With Scaling and Sampling Universe

**Section 1: Values surrounding logistics of SRA policy**		
**Section Scaling**	10-point Likert: **I strongly disagree with this (1)** to **I strongly agree with this (10)** or I'd prefer not to answer	
**Universe**	**Question Prompt Text**	**Proportion not answered**
**HCP Header**	*If there are not enough ventilators or other critical care resources for everyone during a crisis, hospitals and health care workers may need to make decisions about who to give those resources to first.*<br><br>*Again, this would be a crisis so catastrophic that providing usual or standard levels of care to all patients is no longer possible, and there aren't enough critical care resources (such as ventilators, dialysis machines, ECMO circuits, other supplies, medications, or staff) to care for everyone who needs these resources, and some people will die without these resources.*<br><br>*Please tell us how you feel hospitals and health care workers should make decisions like these in general.*	---
**Non-HCP Header**	*If there are not enough ventilators or other critical care resources for everyone during a crisis, hospitals and health care workers may need to make decisions about who to give those resources to first.*<br><br>*Again, this would be a crisis so catastrophic that providing usual or standard levels of care to all patients is no longer possible, and there aren't enough critical care resources (such as ventilators, dialysis machines, ECMO circuits, other supplies, medications, or staff) to care for everyone who needs these resources, and some people will die without these resources.*<br><br>*Please tell us how you feel hospitals and health care workers should make decisions like these in general.*	---
**ALL**	They should try to save the most number of lives possible	1.4%
**ALL**	They should take life support away from some patients in order to give it to other patients who are more likely to survive	5.9%
**ALL**	They should make decisions on a first-come, first-served basis	3.8%
**ALL**	They should apply the same rules to decide who gets life support to all patients equally	2.9%
**ALL**	The same rules should apply to all patients even if they were admitted to the hospital before the crisis started	4.5%
**ALL**	The same rules should apply to all patients even if they're in the hospital for reasons that aren't related to the disaster or pandemic	4.1%
**ALL**	Hospital committees (instead of individual doctors) should make these decisions	4.7%
**ALL**	Hospital committees should not know the identities of the patients and use only medical information to make decisions	3.3%
**ALL**	Policies like this should be developed with input from patients and community members	4.9%
**HCP Only**	Individual doctors should make decisions on a case-by-case basis	2.7%
**HCP Only**	State or federal officials should ensure the law protects hospitals and health care providers who use these policies during a crisis from civil lawsuits (such as malpractice claims)	2.9%

© 2024 Buhr RG et al. *JAMA Network Open.*

**HCP Only**	State or federal officials should ensure the law protects hospitals and health care providers who use these policies during a crisis from criminal penalties (such as criminal negligence claims)	3.5%
**Non-HCP only**	State or federal officials should ensure the law protects hospitals and health care providers who use these policies during a crisis from legal repercussions (like malpractice or negligence claims)	6.5%
**Section 2: Values surrounding decision making around SRA policy**		
**Section Scaling**	10-point Likert: **I strongly disagree with this (1)** to **I strongly agree with this (10)** or I'd prefer not to answer	
**Universe**	**Question Prompt Text**	**Proportion not answered**
**HCP Header**	How strongly do you agree or disagree with the following statements about who should be responsible for making policies to determine who gets life support in a crisis?	---
HCP Only	There should not be policies like this at all	2.1%
HCP Only	Individual hospitals or health systems	1.9%
HCP Only	Health insurers	1.9%
HCP Only	Local (city or county) governments	1.9%
HCP Only	State governments	1.9%
HCP Only	The federal government	1.6%
HCP Only	Medical professional societies (for example: American Medical Association, nursing unions, specialty societies)	3.1%
**Section 3: Values surrounding health factors considered in SRA policy**		
**Section Scaling**	9-point Likert: **Should be much less likely to get life support (1)** to **Should not influence one way or the other (5)** to **Should be much more likely to get life support (9)** or I'd prefer not to answer	
**Universe**	**Question Prompt Text**	**Proportion not answered**
**HCP Header**	How strongly do you feel the following health factors should influence how hospitals and health care workers decide who receives a ventilator or other critical care resources during a crisis like a disaster or pandemic?<br><br>Again, as a reminder, this would be a crisis so catastrophic that providing usual or standard levels of care to all patients is no longer possible, and there aren't enough critical care resources (such as ventilators, dialysis machines, ECMO circuits, other supplies, medications, or staff) to care for everyone who needs these resources, and some people will die without these resources.	---
**Non-HCP Header**	*How strongly do you feel the following health factors should influence how hospitals and health care workers decide who receives life support during a crisis like a disaster or pandemic?*<br><br>*Again, as a reminder, this would be a crisis so catastrophic that providing usual or standard levels of care to all patients is no longer possible, and*	---

© 2024 Buhr RG et al. *JAMA Network Open.*

	there aren't enough critical care resources (such as life support machines, supplies, medications, or staff) to care for everyone who needs these resources, and some people might die without these resources.	
ALL	Patients who are deemed less likely to survive and make it out of the hospital alive	4.3%
ALL	Patients who have physical or intellectual disabilities	3.4%
ALL	Patients who have shorter expected lifespans because of chronic illness	4.1%
ALL	Patients who are elderly	3.7%
ALL	Patients who are children	2.7%
ALL	Patients expected to have a poor quality of life if they survive	3.8%
ALL	Patients expected to need life support for a long time to recover from their illness	4.0%
HCP Only	Patients who are chronically dependent on ventilators	4.1%
HCP Only	Patients in persistent vegetative or minimally conscious states	2.5%
**Section 4: Values surrounding social or non-health factors considered in SRA policy**		
**Section Scaling**	9-point Likert: **Should be much less likely to get life support (1)** to **Should not influence one way or the other (5)** to **Should be much more likely to get life support (9)** or I'd prefer not to answer	
**Universe**	**Question Prompt Text**	**Proportion not answered**
**HCP Header**	*How strongly do you agree or disagree about these statements about factors not related to a patient's health should be considered when making decisions about who should receive ventilators or other critical care resources during a crisis like a disaster of pandemic?*<br><br>*Again, as a reminder, this would be a crisis so catastrophic that providing usual or standard levels of care to all patients is no longer possible, and there aren't enough critical care resources (such as ventilators, dialysis machines, ECMO circuits, other supplies, medications, or staff) to care for everyone who needs these resources, and some people will die without these resources.*	---
**Non-HCP Header**	*How strongly do you agree or disagree about these statements about factors not related to a patient's health should be considered when making decisions about who should receive life support during a crisis like a disaster of pandemic?*<br><br>*Again, as a reminder, this would be a crisis so catastrophic that providing usual or standard levels of care to all patients is no longer possible, and there aren't enough critical care resources (such as life support machines, supplies, medications, or staff) to care for everyone who needs these resources, and some people might die without these resources.*	---
ALL	People who are wealthy, famous, or in positions of power (for example: celebrities or politicians)	1.5%
ALL	People who are a racial or ethnic minority	1.6%
ALL	People who are LGBTQ+ (e.g, lesbian, gay, bisexual, or transgender)	1.8%
ALL	People who are prisoners	2.1%
ALL	People without health insurance	1.6%

© 2024 Buhr RG et al. *JAMA Network Open.*

ALL	People who are undocumented immigrants	2.0%
ALL	Patients who have shorter expected lifespans because of a serious health condition even if that condition is more common among people with a disability	3.2%
ALL	Patients who have shorter expected lifespans because of a serious health condition even if that condition is more common among racial or ethnic minorities	2.9%
ALL	Patients who have shorter expected lifespans because of a serious health condition even if that condition is more common among people living in poverty	2.8%
HCP Only	People who are philanthropic donors to the hospital or health system	1.2%
	**Section 5: Values surrounding exemptions to SRA policy**	
**Section Scaling**	9-point Likert: **Should be much less likely to get life support (1)** to **Should not influence one way or the other (5)** to **Should be much more likely to get life support (9)** or I'd prefer not to answer	
**Universe**	**Question Prompt Text**	**Proportion not answered**
**HCP Header**	There may be some situations where exceptions are made for certain groups of people under policies like this. How strongly do you agree or disagree that some individuals should be more likely to receive ventilators or other critical care during a crisis because of certain factors not necessarily related to their health?<br><br>Again, as a reminder, this would be a crisis so catastrophic that providing usual or standard levels of care to all patients is no longer possible, and there aren't enough critical care resources (such as ventilators, dialysis machines, ECMO circuits, other supplies, medications, or staff) to care for everyone who needs these resources, and some people will die without these resources.	---
**Non-HCP Header**	*There may be some situations where exceptions are made for certain groups of people under policies like this. How strongly do you agree or disagree that some individuals should be more likely to receive life support or critical care during a crisis because of certain factors not necessarily related to their health?*<br><br>*Again, as a reminder, this would be a crisis so catastrophic that providing usual or standard levels of care to all patients is no longer possible, and there aren't enough critical care resources (such as life support machines, supplies, medications, or staff) to care for everyone who needs these resources, and some people might die without these resources.*	---
ALL	Patients who are pregnant in the first trimester	2.8%
ALL	Patients who are pregnant in the third trimester	2.4%
ALL	First responders (for example: police, fire fighters)	1.9%
ALL	Health care workers in general who are critical to caring for patients	1.9%
ALL	Health care workers specifically who are on the front lines and at increased risk of harm from the pandemic	1.6%
ALL	Patients who are participating in medical research studies	2.0%
ALL	Patients who are the sole or only caregiver of a family member (for example: a child or a disabled or elderly relative)	2.3%
ALL	Members of the military or veterans	1.8%
ALL	Public officials (for example: a mayor, governor, president, or congressperson)	2.2%

© 2024 Buhr RG et al. *JAMA Network Open.*

ALL	Patients who are on the list to get an organ transplant	3.2%
ALL	Patients who recently received an organ transplant	3.4%
HCP Only	Patients who have recently undergone major surgery (not related to a transplant)	2.9%
HCP Only	Patients who have had a complication from medical care (for example: a procedural or surgical complication or adverse reaction to a medication)	2.8%
HCP Only	Families or friends of critical health workers	2.1%

© 2024 Buhr RG et al. *JAMA Network Open.*

eFigure 1. Values for SRA Policies Related to Social Factors (Unimputed)

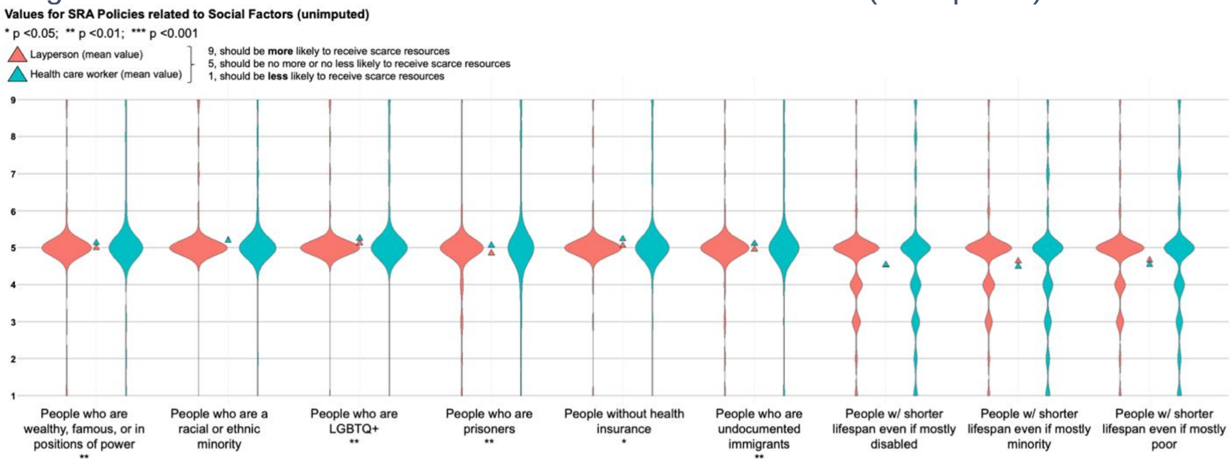

eFigure 1: Violin plot of responses to SRA social factor questions by health care worker status. Plots demonstrate distribution of responses. The teal strips represent self-identified health care workers while the coral strips represent laypersons, with the triangles indicating mean values.

© 2024 Buhr RG et al. *JAMA Network Open.*

eFigure 2. Values for Use of Exemptions in SRA Design

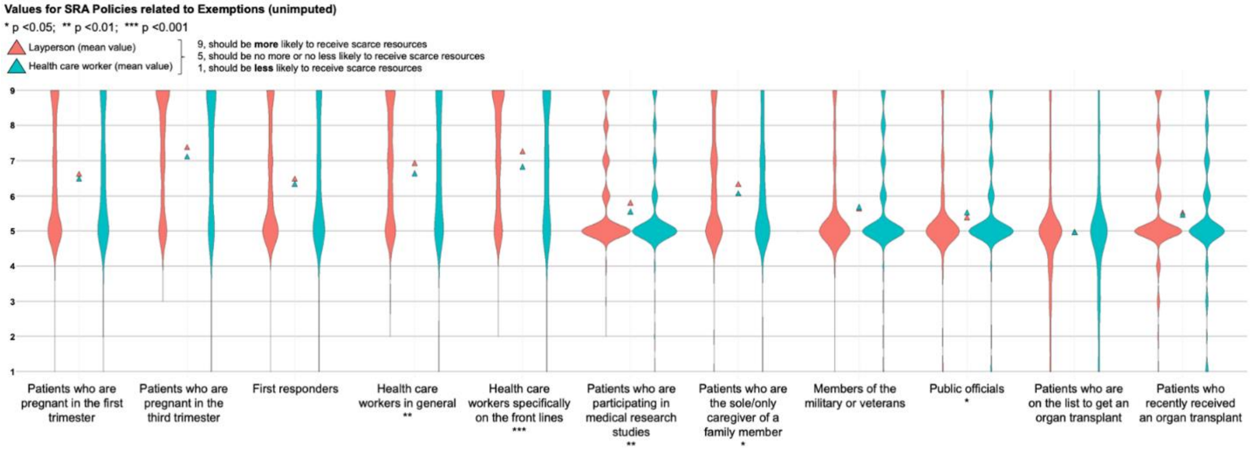

eFigure 2: Violin plot of responses to SRA exemption questions by health care worker status. Plots demonstrate distribution of responses. The teal strips represent self-identified health care workers while the coral strips represent laypersons, with the triangles representing mean values.

© 2024 Buhr RG et al. *JAMA Network Open.*

eTable 2. Response Scores Comparing Layperson and HCP by Item, Unimputed Responses

	Lay		HCP		
	Mean	SD	Mean	SD	p-value
Values - Logistics (1, strongly disagree to 10, strongly agree)					
**Try to save the most number of lives possible**	9.05	1.90	8.76	2.16	**0.031**
**Take life support away from some Patients to give to others**	5.40	2.74	6.41	2.71	**<.001**
**Make decisions on a first-come, first-served basis**	3.90	2.69	4.20	3.00	0.258
**Apply the same rules to all patients equally**	7.75	2.72	7.82	2.57	0.945
**Same rules even if patient admitted before the crisis**	7.60	2.71	7.59	2.68	0.778
**Same rules even if patient hospitalized unrelated to pandemic**	7.80	2.61	7.59	2.71	0.101
**Hospital committees should make these decisions**	5.82	3.09	6.43	2.85	**0.001**
**Hospital committees should not know patient identities**	8.43	2.35	8.28	2.45	0.186
**Should be developed with patients and community**	7.10	2.91	6.74	3.10	0.056
Values - Health Factors (1, should be less likely to 9, should be more likely)					
**Patients who are deemed less likely to survive**	3.71	1.87	3.38	2.24	**<.001**
**Patients who have physical or intellectual disabilities**	5.07	1.09	5.18	1.40	0.392
**Patients with shorter expected lifespans b/c chronic illness**	4.45	1.41	4.27	1.93	**<.001**
**Patients who are elderly**	4.70	1.47	4.75	1.68	0.534
**Patients who are children**	7.20	1.79	7.08	1.89	0.342
**Patients expected to have a poor quality of life if they survive**	4.02	1.53	3.70	2.00	**<.001**
**Patients needing life support for a long time to recover**	4.71	1.37	4.98	1.82	**0.022**
Values - Social Factors (1, should be less likely to 9, should be more likely)					
**People who are wealthy, famous, or in positions of power**	5.00	1.00	5.14	1.16	**0.005**
**People who are a racial or ethnic minority**	5.21	0.88	5.20	1.06	0.472
**People who are LGBTQ+**	5.13	0.86	5.26	1.11	**0.010**
**People who are prisoners**	4.86	1.05	5.07	1.36	**0.003**
**People without health insurance**	5.06	0.88	5.24	1.16	**0.014**
**People who are undocumented immigrants**	4.96	1.00	5.12	1.25	**0.003**
**People w/ shorter lifespan even if mostly disabled**	4.54	1.22	4.54	1.71	0.311
**People w/ shorter lifespan even if mostly minority**	4.64	1.28	4.50	1.68	0.052
**People w/ shorter lifespan even if mostly poor**	4.67	1.23	4.55	1.69	0.070
Values - Exemptions (1, should be less likely to 9, should be more likely)					
**Patients who are pregnant in the first trimester**	6.61	1.69	6.47	1.70	0.341
**Patients who are pregnant in the third trimester**	7.37	1.65	7.10	1.91	0.052
**First responders**	6.46	1.63	6.32	1.76	0.331
**Health care workers in general**	6.92	1.64	6.62	1.76	**0.009**
**Health care workers specifically on the front lines**	7.25	1.64	6.81	1.88	**<.001**
**Patients who are participating in medical research studies**	5.79	1.36	5.53	1.33	**0.001**
**Patients who are the sole/only caregiver of a family member**	6.32	1.53	6.05	1.53	**0.015**

© 2024 Buhr RG et al. *JAMA Network Open.*

	Lay		HCP		
	Mean	SD	Mean	SD	p-value
**Members of the military or veterans**	5.62	1.34	5.66	1.38	0.112
**Public officials**	5.37	1.25	5.50	1.30	**0.016**
**Patients who are on the list to get an organ transplant**	4.96	1.50	4.95	1.60	0.618
**Patients who recently received an organ transplant**	5.51	1.47	5.45	1.67	0.533
Preferences - Disclosure (1, strongly disagree to 10, strongly agree)					
**Hospitals should make this information public**	8.42	2.33	8.09	2.49	**0.008**
**Hospitals should tell Patients only if admitted in critical condition**	5.08	3.51	4.96	3.23	0.563
**I would consider policies when deciding if I would go**	7.71	2.70	7.41	2.65	**0.018**
Trust (1, strongly disagree to 10, strongly agree)					
**Hospitals/doctors will apply in a fair and consistent way**	6.25	2.63	6.42	2.59	0.253
**Hospitals/doctors to be honest and transparent**	6.16	2.64	6.45	2.62	0.054
**Anxious or worried when I think about these policies**	6.46	2.87	6.40	2.94	0.863
**My doctors to be honest with me about survival chances**	7.81	2.17	7.58	2.29	0.086

© 2024 Buhr RG et al. *JAMA Network Open.*

eTable 3. Nonimputed (All Respondent) vs. Mean Imputed Respondent and Unimputed California-Only Responses for Agreement Ratings With UC SRA Policy Domains as Drafted

	**Total**<br><br>**Mean (95% CI)**	**Lay**<br><br>**Mean (95% CI)**	**HCP**<br><br>**Mean (95% CI)**	**Lay vs HCP P-Value**
**As observed / non-imputed SRA**				
Value - Logistics (% agreement)	71.3% (70.6%-72.1%)	71.1% (70.2%-72.0%)	71.9% (70.5%-73.4%)	0.3384
Value - Health (% agreement)	70.3% (69.4%-71.1%)	71.7% (70.8%-72.7%)	66.9% (65.1%-68.6%)	**<0.0001**
Value - Social (% agreement)	91.1% (90.0%-92.2%)	92.5% (91.3%-93.6%)	87.9% (85.6%-90.2%)	**<0.0001**
Value - Exemptions (% agreement)	71.0% (70.4%-71.7%)	71.9% (71.1%-72.6%)	69.1% (67.7%-70.5%)	**0.0002**
All Value Items (% agreement)	76.0% (75.5%-76.5%)	76.8% (76.3%-77.3%)	74.2% (73.1%-75.4%)	**<0.0001**
**Imputed SRA**				
Value - Logistics (% agreement)	70.8% (70.1%-71.6%)	70.5% (69.6%-71.4%)	71.6% (70.1%-73.1%)	0.1719
Value - Health (% agreement)	69.8% (69.0%-70.7%)	71.2% (70.2%-72.1%)	66.6% (64.8%-68.4%)	**<0.0001**
Value - Social (% agreement)	90.6% (89.5%-91.7%)	92.0% (90.8%-93.1%)	87.3% (85.0%-89.6%)	**<0.0001**
Value - Exemptions (% agreement)	70.8% (70.1%-71.5%)	71.6% (70.8%-72.4%)	69.0% (67.6%-70.5%)	**0.0005**
All Value Items (% agreement)	75.6% (75.0%-76.1%)	76.3% (75.7%-76.8%)	74.0% (72.8%-75.1%)	**<0.0001**
**Unimputed, California only**				
Value - Logistics (% agreement)	72.1% (71.2%-73.0%)	71.3% (70.2%-73.3%)	74.1% (72.5%-75.8%)	**0.0043**
Value - Health (% agreement)	72.3% (71.5%-73.2%)	72.3% (71.8%-73.8%)	71.1% (69.4%-72.9%)	**0.0801**
Value - Social (% agreement)	84.4% (83.5%-85.3%)	84.0% (83.0%-85.1%)	85.3% (83.7%-87.0%)	0.1860
Value - Exemptions (% agreement)	71.7% (70.9%-72.5%)	72.4% (71.5%-73.3%)	70.0% (67.4%-71.7%)	**0.0079**
All Value Items (% agreement)	75.6% (75.4%-76.4%)	75.7% (75.1%-76.3%)	76.1% (75.2%-77.2%)	**0.4242**

© 2024 Buhr RG et al. *JAMA Network Open.*

eTable 4. Response Scores Comparing Layperson and HCP by Item, Restricted to California Respondents

	Lay		HCP		
	Mean	SD	Mean	SD	p-value
Values - Logistics (1, strongly disagree to 10, strongly agree)					
**Try to save the most number of lives possible**	9.07	1.94	9.12	1.85	0.5040
**Take life support away from some Patients to give to others**	5.33	2.73	6.36	2.73	<.0001
**Make decisions on a first-come, first-served basis**	3.71	2.59	3.85	2.85	0.8650
**Apply the same rules to all patients equally**	7.81	2.72	8.03	2.45	0.4716
**Same rules even if patient admitted before the crisis**	7.65	2.68	7.79	2.63	0.4391
**Same rules even if patient hospitalized unrelated to pandemic**	7.81	2.63	7.85	2.62	0.9819
**Hospital committees should make these decisions**	5.75	3.11	6.43	2.86	0.0027
**Hospital committees should not know patient identities**	8.43	2.40	8.51	2.38	0.7842
**Should be developed with patients and community**	7.21	2.90	6.72	3.12	0.0288
Values - Health Factors (1, should be less likely to 9, should be more likely)					
**Patients who are deemed less likely to survive**	3.64	1.78	3.08	1.97	<.0001
**Patients who have physical or intellectual disabilities**	5.03	0.97	4.96	1.11	0.1866
**Patients with shorter expected lifespans b/c chronic illness**	4.41	1.39	3.96	1.61	<.0001
**Patients who are elderly**	4.60	1.40	4.57	1.47	0.1970
**Patients who are children**	7.23	1.79	7.19	1.81	0.6370
**Patients expected to have a poor quality of life if they survive**	3.96	1.50	3.40	1.69	<.0001
**Patients needing life support for a long time to recover**	4.68	1.33	4.74	1.60	0.7425
Values - Social Factors (1, should be less likely to 9, should be more likely)					
**People who are wealthy, famous, or in positions of power**	4.94	0.92	5.05	0.84	0.0183
**People who are a racial or ethnic minority**	5.19	0.84	5.09	0.58	0.4139
**People who are LGBTQ+**	5.12	0.85	5.13	0.70	0.5597
**People who are prisoners**	4.84	1.01	4.83	1.10	0.7238
**People without health insurance**	5.04	0.85	5.06	0.78	0.8824
**People who are undocumented immigrants**	4.97	0.89	5.02	0.89	0.3815
**People w/ shorter lifespan even if mostly disabled**	4.50	1.19	4.37	1.43	0.0333
**People w/ shorter lifespan even if mostly minority**	4.60	1.23	4.37	1.43	0.0188
**People w/ shorter lifespan even if mostly poor**	4.62	1.18	4.34	1.42	0.0010
Values - Exemptions (1, should be less likely to 9, should be more likely)					
**Patients who are pregnant in the first trimester**	6.54	1.70	6.45	1.71	0.6119
**Patients who are pregnant in the third trimester**	7.36	1.66	7.28	1.75	0.6421
**First responders**	6.48	1.65	6.32	1.63	0.2549
**Health care workers in general**	6.93	1.65	6.65	1.71	0.0320
**Health care workers specifically on the front lines**	7.30	1.64	6.91	1.75	0.0024
**Patients who are participating in medical research studies**	5.78	1.35	5.47	1.17	0.0009

© 2024 Buhr RG et al. *JAMA Network Open.*

	Lay		HCP		
	**Mean**	**SD**	**Mean**	**SD**	**p-value**
**Patients who are the sole/only caregiver of a family member**	6.33	1.53	6.03	1.42	0.0087
**Members of the military or veterans**	5.58	1.29	5.57	1.19	0.2134
**Public officials**	5.33	1.22	5.46	1.18	0.0696
**Patients who are on the list to get an organ transplant**	4.90	1.46	4.73	1.39	0.3454
**Patients who recently received an organ transplant**	5.48	1.44	5.33	1.50	0.8356

© 2024 Buhr RG et al. *JAMA Network Open.*

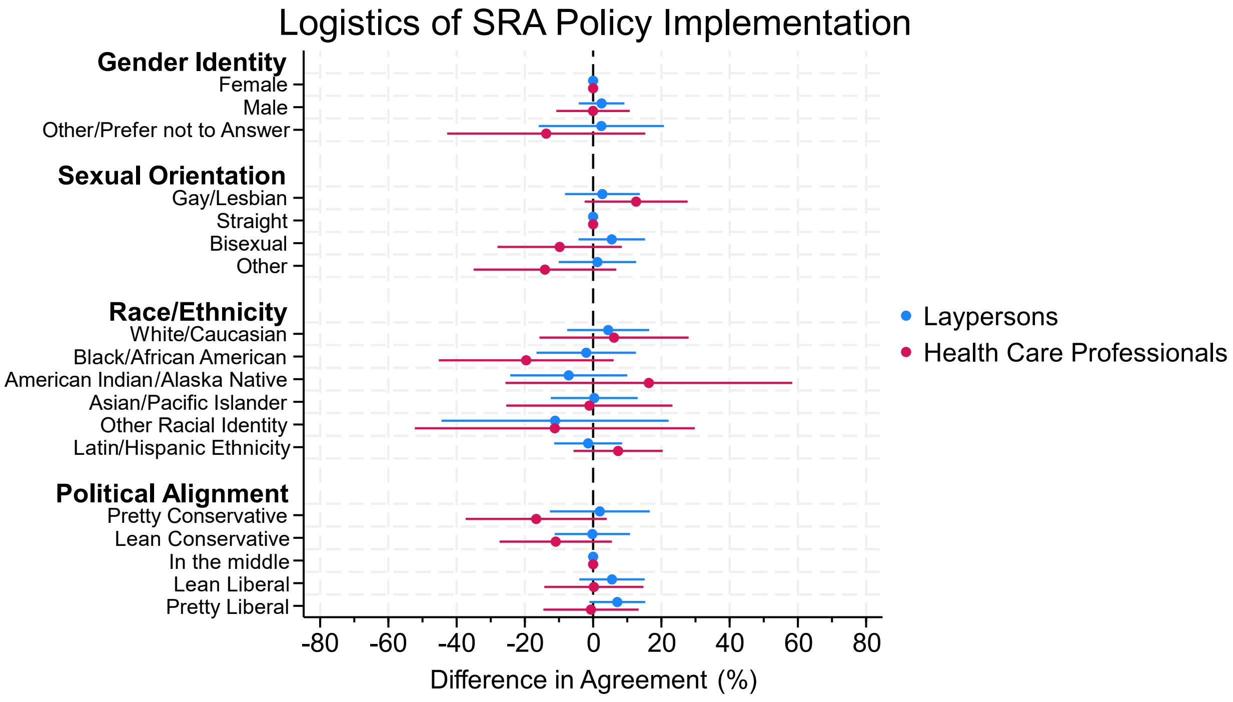

eFigure 3. Differences in Agreement With Logistics of SRA Policy Implementation by Respondent Characteristics, Stratified by HCP Status. Fractional probit model additionally controlling for age, marital status, employment, education, households with children, and essential worker status.

© 2024 Buhr RG et al. *JAMA Network Open.*

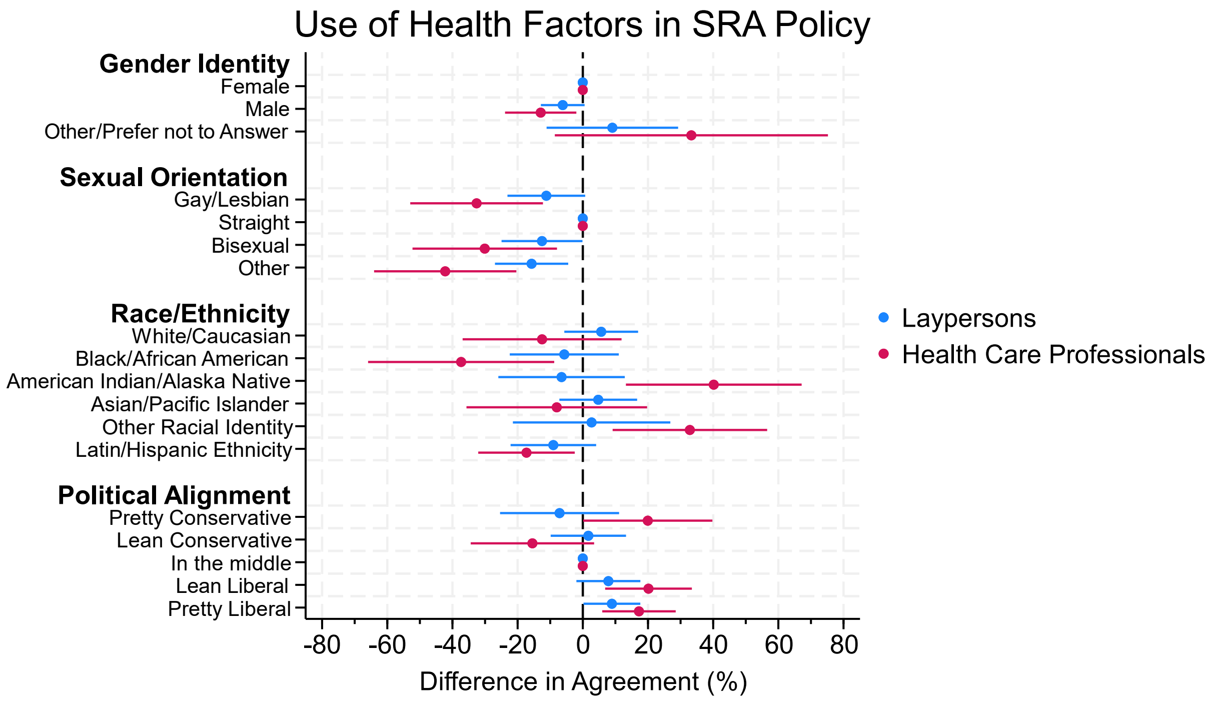

eFigure 4. Differences in Agreement With Use of Health Factors in SRA Policy Design by Respondent Characteristics, Stratified by HCP Status. Fractional probit model additionally controlling for age, marital status, employment, education, households with children, and essential worker status.

© 2024 Buhr RG et al. *JAMA Network Open.*

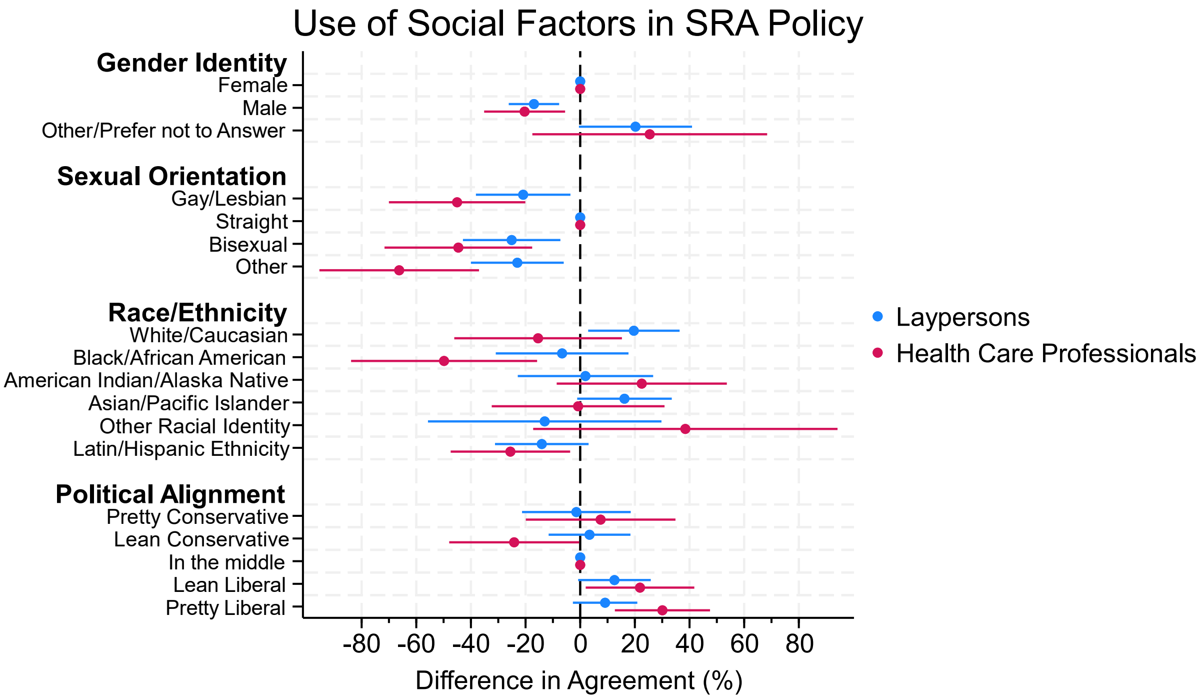

eFigure 5. Differences in Agreement With Use of Nonhealth Social Factors in SRA Policy Design by Respondent Characteristics, Stratified by HCP Status. Fractional probit model additionally controlling for age, marital status, employment, education, households with children, and essential worker status.

© 2024 Buhr RG et al. *JAMA Network Open.*

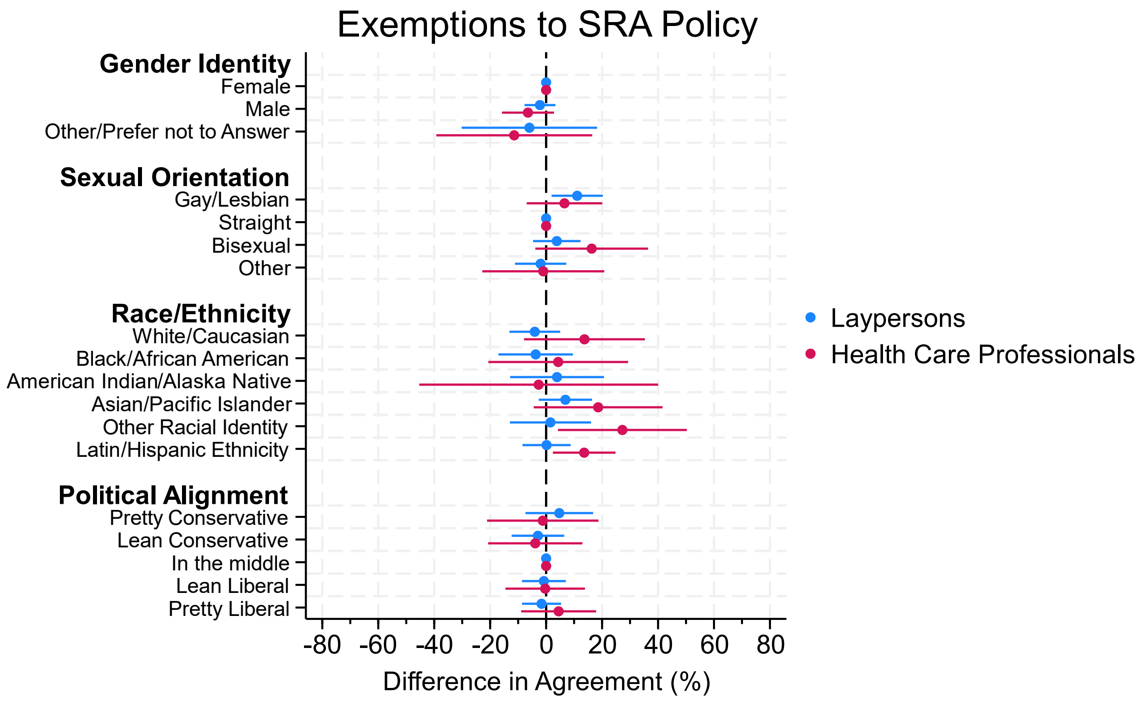

eFigure 6. Differences in Agreement With Exemptions to SRA Policy by Respondent Characteristics, Stratified by HCP Status. Fractional probit model additionally controlling for age, marital status, employment, education, households with children, and essential worker status

© 2024 Buhr RG et al. *JAMA Network Open.*
