# Supplementary material for: Health Professional vs Layperson Values and Preferences on Scarce Resource Allocation
Source: JAMA Netw Open. 2024 Mar 12;7(3):e241958. doi: 10.1001/jamanetworkopen.2024.1958 (PMC10933708; doi:10.1001/jamanetworkopen.2024.1958)
Supplement: Supplement 2. — Data Sharing Statement [file jamanetwopen-e241958-s002.pdf]

# Data Sharing Statement

Buhr. Health Professional vs Layperson Values and Preferences on Scarce Resource Allocation. *JAMA Netw Open*. Published March 12, 2024.
doi:10.1001/jamanetworkopen.2024.1958

**Data**
**Data available:** Yes
**Data types:** Deidentified participant data, Data dictionary
**How to access data:** Data will be made available upon reasonable request and executed data use agreement with the authors.
**When available:** With publication

**Supporting Documents**
**Document types:** None

**Additional Information**
**Who can access the data:** Researchers whose proposed use of data has been approved.
**Types of analyses:** For research analyses
**Mechanisms of data availability:** With investigator support after approval of a proposal and a signed data use agreement.
**Any additional restrictions:** N/A
